# Supplementary material for: FexSplice: A LightGBM-Based Model for Predicting the Splicing Effect of a Single Nucleotide Variant Affecting the First Nucleotide G of an Exon
Source: Genes (Basel). 2023 Sep 6;14(9):1765. doi: 10.3390/genes14091765 (PMC10531444; doi:10.3390/genes14091765)

Supplementary Figure S1. Heatmap of Spearman's correlation coefficients of 115 feature values to indicate multiple collinearity of features.

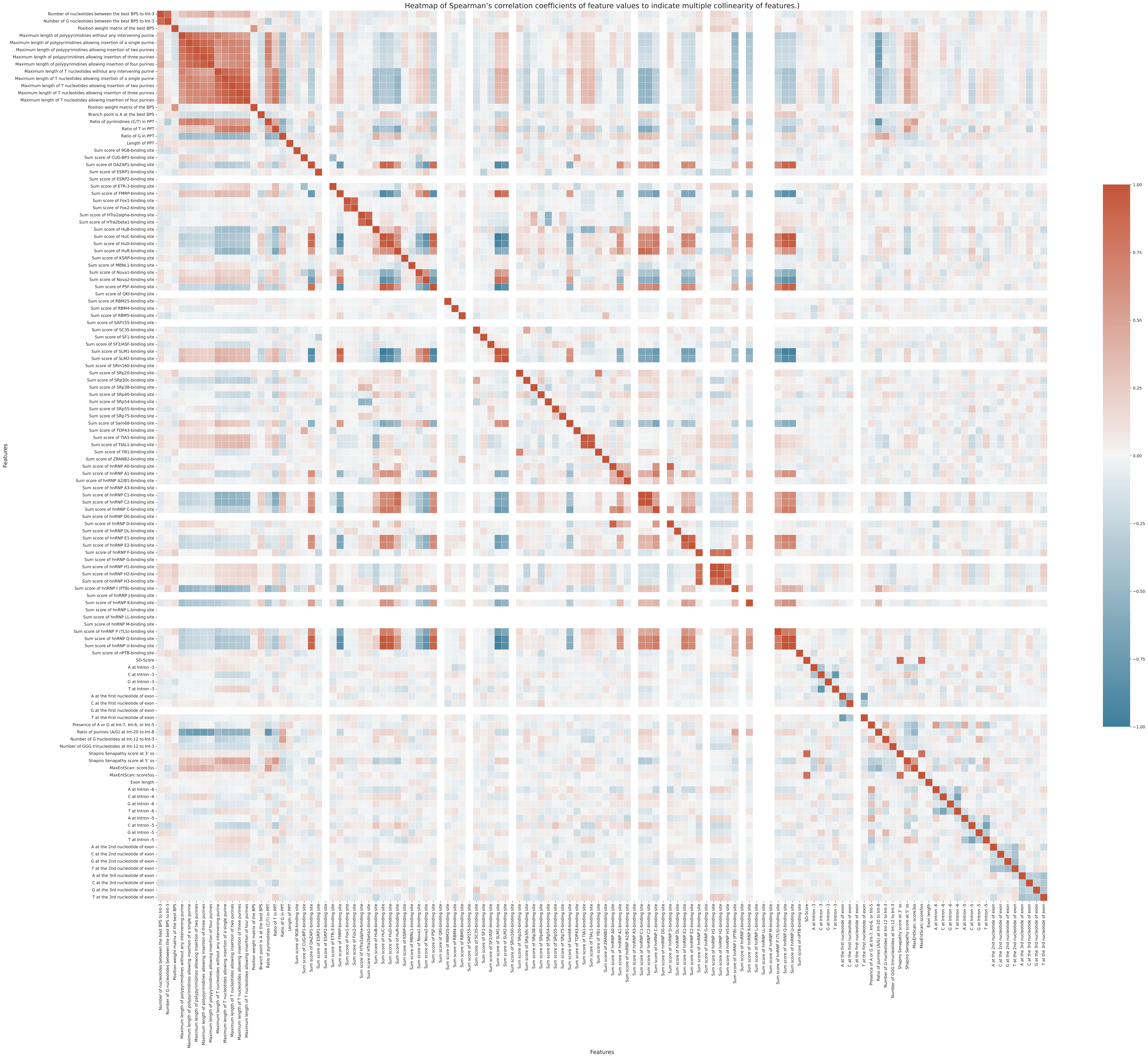

Supplement: Supplementary file 1 [file genes-14-01765-s001.zip › Fig S1.pdf]
