# Supplementary material for: FexSplice: A LightGBM-Based Model for Predicting the Splicing Effect of a Single Nucleotide Variant Affecting the First Nucleotide G of an Exon
Source: Genes (Basel). 2023 Sep 6;14(9):1765. doi: 10.3390/genes14091765 (PMC10531444; doi:10.3390/genes14091765)
Supplement: Supplementary file 1 [file genes-14-01765-s001.zip › Fig S2.pdf]

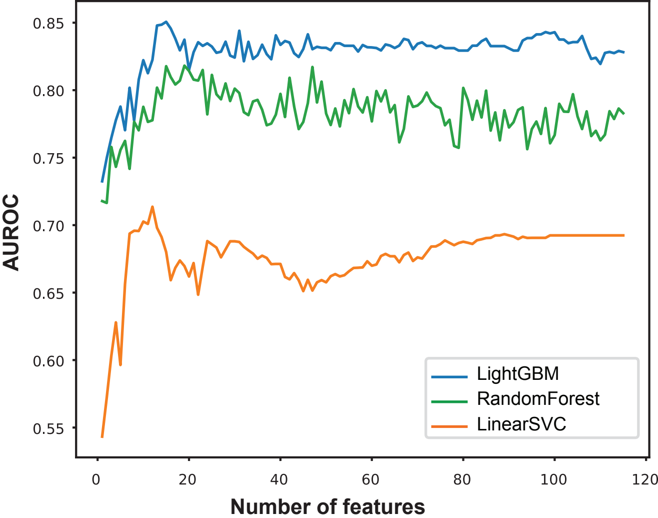

Supplementary Figure S2. Feature elimination of LinearSVC, RandomForst, and LightGBM models. Features were eliminated one-by-one by LOOCV using SelectFromModel. Area-under-the-receiver-operating-characteristics-curve (AUROC) is plotted.
