# Supplementary material for: FexSplice: A LightGBM-Based Model for Predicting the Splicing Effect of a Single Nucleotide Variant Affecting the First Nucleotide G of an Exon
Source: Genes (Basel). 2023 Sep 6;14(9):1765. doi: 10.3390/genes14091765 (PMC10531444; doi:10.3390/genes14091765)
Supplement: Supplementary file 1 [file genes-14-01765-s001.zip › Fig S3.pdf]

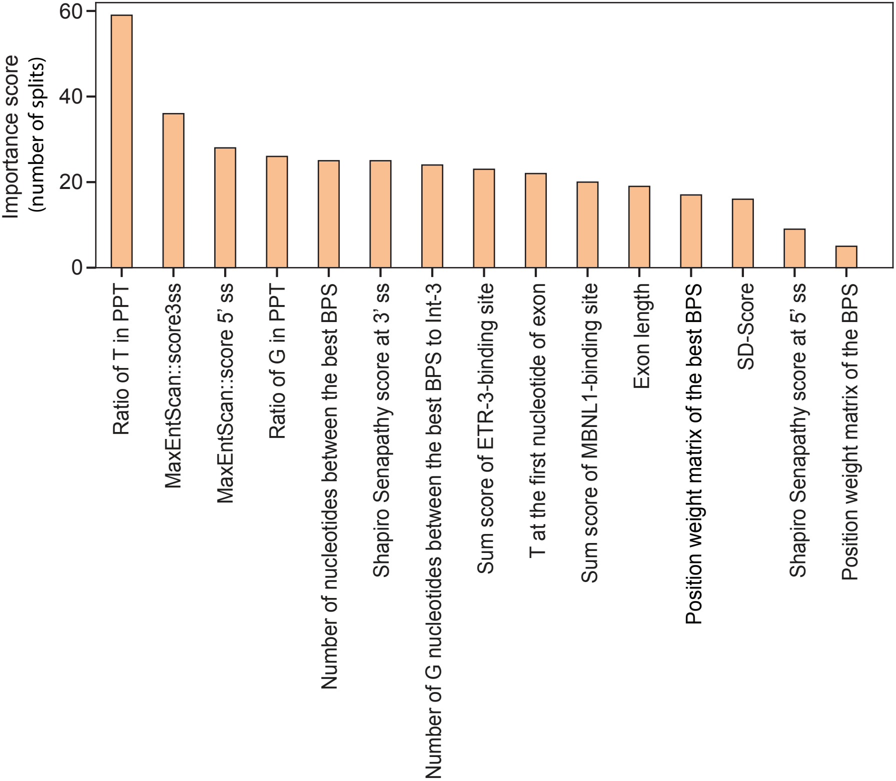

Supplementary Figure S3. Feature importance of 15 feature-based LightGBM models by 10-fold cross-validation. The feature importance is represented by the number of times each feature is used to split the data across all trees in the ensemble. Features with higher importance values have a more significant impact on the model.
