# Supplementary material for: FexSplice: A LightGBM-Based Model for Predicting the Splicing Effect of a Single Nucleotide Variant Affecting the First Nucleotide G of an Exon
Source: Genes (Basel). 2023 Sep 6;14(9):1765. doi: 10.3390/genes14091765 (PMC10531444; doi:10.3390/genes14091765)
Supplement: Supplementary file 1 [file genes-14-01765-s001.zip › Supplementary Table S2.pdf]

Supplementary Table S2. 115 features to dictate the strength of splicing *cis*-elements

Features	3’/Ex/5’	Position[a]
Best-BPS[b]		
Number of nucleotides between the best BPS to Int-3	3'	Int-50 toInt-3
Number of G nucleotides between the best BPS to Int-3	3'	Int-50 to Int-3
Position weight matrix of the best BPS	3'	Int-50 to Int-3
PPT		
Maximum length of polypyrimidines without any intervening purine	3'	Int-50 to Int-3
Best-BPS-PPT[c]		
Position weight matrix of the BPS	3'	Int-50 to Int-3
Branch point is A at the best BPS	3'	Int-50 to Int-3
Ratio of pyrimidines (C/T) in PPT	3'	Int-50 to Int-3
Ratio of T in PPT	3'	Int-50 to Int-3
Ratio of G in PPT	3'	Int-50 to Int-3
Length of PPT	3'	Int-50 to Int-3
Individual nucleotides		
A at Intron -6	3'	Int-6
C at Intron -6	3'	Int-6
G at Intron -6	3'	Int-6
T at Intron -6	3'	Int-6
A at Intron -5	3'	Int-5
C at Intron -5	3'	Int-5
G at Intron -5	3'	Int-5
T at Intron -5	3'	Int-5
A at Intron -3	3'	Int-3
C at Intron -3	3'	Int-3
G at Intron -3	3'	Int-3
T at Intron -3	3'	Int-3
A at the first nucleotide of exon	Ex	Ex+1
C at the first nucleotide of exon	Ex	Ex+1
G at the first nucleotide of exon	Ex	Ex+1
T at the first nucleotide of exon	Ex	Ex+1
A at the 2nd nucleotide of exon	Ex	Ex+2
C at the 2nd nucleotide of exon	Ex	Ex+2
G at the 2nd nucleotide of exon	Ex	Ex+2
T at the 2nd nucleotide of exon	Ex	Ex+2
A at the 3rd nucleotide of exon	Ex	Ex+2
C at the 3rd nucleotide of exon	Ex	Ex+2
G at the 3rd nucleotide of exon	Ex	Ex+2
T at the 3rd nucleotide of exon	Ex	Ex+2
Presence of A or G at Int-7, Int-6, or Int-5	3'	Int-7 to Int-5
Ratio of purines (A/G) at Int-20 to Int-8	3'	Int-20 to Int-8
Number of G nucleotides at Int-12 to Int-3	3'	Int-12 to Int-3
Number of GGG trinucleotides at Int-12 to Int-3	3'	Int-12 to Int-3
Other parameters		
SD-Score	Ex/5'	Ex-3 to Int+6
Exon length	Ex	Ex
MaxEntScan::score3ss	3'/Ex	Int-20 to Ex+3
MaxEntScan::score5ss	Ex/5'	Ex-3 to Int+6
Shapiro Senapathy score at 3’ ss	3’/Ex	Int-14 to Ex+1
Shapiro Senapathy score at 5’ ss	Ex/5’	Ex-2 to Int+6
SpliceAid2 scores of RNA-bindingprotein[d]		
Sum score of 9G8-binding site	3’/Ex/5’	Int-50 to Int+50

Sum score of CUG-BP1-binding site	3’/Ex/5’	Int-50 to Int+50
Sum score of DAZAP1-binding site	3’/Ex/5’	Int-50 to Int+50
Sum score of ESRP1-binding site	3’/Ex/5’	Int-50 to Int+50
Sum score of ESRP2-binding site	3’/Ex/5’	Int-50 to Int+50
Sum score of ETR-3-binding site	3’/Ex/5’	Int-50 to Int+50
Sum score of FMRP-binding site	3’/Ex/5’	Int-50 to Int+50
Sum score of Fox1-binding site	3’/Ex/5’	Int-50 to Int+50
Sum score of Fox2-binding site	3’/Ex/5’	Int-50 to Int+50
Sum score of HTra2alpha-binding site	3’/Ex/5	Int-50 to Int+50
Sum score of HTra2beta1-binding site	3’/Ex/5’	Int-50 to Int+50
Sum score of HuB-binding site	3’/Ex/5’	Int-50 to Int+50
Sum score of HuC-binding site	3’/Ex/5’	Int-50 to Int+50
Sum score of HuD-binding site	3’/Ex/5’	Int-50 to Int+50
Sum score of HuR-binding site	3’/Ex/5’	Int-50 to Int+50
Sum score of KSRP-binding site	3’/Ex/5’	Int-50 to Int+50
Sum score of MBNL1-binding site	3’/Ex/5’	Int-50 to Int+50
Sum score of Nova1-binding site	3’/Ex/5’	Int-50 to Int+50
Sum score of Nova2-binding site	3’/Ex/5’	Int-50 to Int+50
Sum score of PSF-binding site	3’/Ex/5’	Int-50 to Int+50
Sum score of QKI-binding site	3’/Ex/5’	Int-50 to Int+50
Sum score of RBM25-binding site	3’/Ex/5’	Int-50 to Int+50
Sum score of RBM4-binding site	3’/Ex/5’	Int-50 to Int+50
Sum score of RBM5-binding site	3’/Ex/5’	Int-50 to Int+50
Sum score of SAP155-binding site	3’/Ex/5’	Int-50 to Int+50
Sum score of SC35-binding site	3’/Ex/5’	Int-50 to Int+50
Sum score of SF1-binding site	3’/Ex/5’	Int-50 to Int+50
Sum score of SF2/ASF-binding site	3’/Ex/5’	Int-50 to Int+50
Sum score of SLM1-binding site	3’/Ex/5’	Int-50 to Int+50
Sum score of SLM2-binding site	3’/Ex/5’	Int-50 to Int+50
Sum score of SRm160-binding site	3’/Ex/5’	Int-50 to Int+50
Sum score of SRp20-binding site	3’/Ex/5’	Int-50 to Int+50
Sum score of SRp30c-binding site	3’/Ex/5’	Int-50 to Int+50
Sum score of SRp38-binding site	3’/Ex/5’	Int-50 to Int+50
Sum score of SRp40-binding site	3’/Ex/5’	Int-50 to Int+50
Sum score of SRp54-binding site	3’/Ex/5’	Int-50 to Int+50
Sum score of SRp55-binding site	3’/Ex/5’	Int-50 to Int+50
Sum score of SRp75-binding site	3’/Ex/5’	Int-50 to Int+50
Sum score of Sam68-binding site	3’/Ex/5’	Int-50 to Int+50
Sum score of TDP43-binding site	3’/Ex/5’	Int-50 to Int+50
Sum score of TIA1-binding site	3’/Ex/5’	Int-50 to Int+50
Sum score of TIAL1-binding site	3’/Ex/5’	Int-50 to Int+50
Sum score of YB1-binding site	3’/Ex/5’	Int-50 to Int+50
Sum score of ZRANB2-binding site	3’/Ex/5’	Int-50 to Int+50
Sum score of hnRNP A0-binding site	3’/Ex/5’	Int-50 to Int+50
Sum score of hnRNP A1-binding site	3’/Ex/5’	Int-50 to Int+50
Sum score of hnRNP A2/B1-binding site	3’/Ex/5’	Int-50 to Int+50
Sum score of hnRNP A3-binding site	3’/Ex/5’	Int-50 to Int+50
Sum score of hnRNP C1-binding site	3’/Ex/5’	Int-50 to Int+50
Sum score of hnRNP C2-binding site	3’/Ex/5’	Int-50 to Int+50
Sum score of hnRNP C-binding site	3’/Ex/5’	Int-50 to Int+50
Sum score of hnRNP D0-binding site	3’/Ex/5’	Int-50 to Int+50
Sum score of hnRNP D-binding site	3’/Ex/5’	Int-50 to Int+50
Sum score of hnRNP DL-binding site	3’/Ex/5’	Int-50 to Int+50
Sum score of hnRNP E1-binding site	3’/Ex/5’	Int-50 to Int+50
Sum score of hnRNP E2-binding site	3’/Ex/5’	Int-50 to Int+50

Sum score of hnRNP F-binding site	3'/Ex/5'	Int-50 to Int+50
Sum score of hnRNP G-binding site	3'/Ex/5'	Int-50 to Int+50
Sum score of hnRNP H1-binding site	3'/Ex/5'	Int-50 to Int+50
Sum score of hnRNP H2-binding site	3'/Ex/5'	Int-50 to Int+50
Sum score of hnRNP H3-binding site	3'/Ex/5'	Int-50 to Int+50
Sum score of hnRNP I (PTB)-binding site	3'/Ex/5'	Int-50 to Int+50
Sum score of hnRNP J-binding site	3'/Ex/5'	Int-50 to Int+50
Sum score of hnRNP K-binding site	3'/Ex/5'	Int-50 to Int+50
Sum score of hnRNP L-binding site	3'/Ex/5'	Int-50 to Int+50
Sum score of hnRNP LL-binding site	3'/Ex/5'	Int-50 to Int+50
Sum score of hnRNP M-binding site	3'/Ex/5'	Int-50 to Int+50
Sum score of hnRNP P (TLS)-binding site	3'/Ex/5'	Int-50 to Int+50
Sum score of hnRNP Q-binding site	3'/Ex/5'	Int-50 to Int+50
Sum score of hnRNP U-binding site	3'/Ex/5'	Int-50 to Int+50
Sum score of nPTB-binding site	3'/Ex/5'	Int-50 to Int+50

[a]The feature was applied to the indicated position. Int+N and Int-N represent the number of intronic nucleotides from the 5' and 3' ss, respectively. Similarly, Ex+N and Ex-N represent the number of exonic nucleotides from the 3' and 5' ss, respectively.

[b]Best-BPS was determined in each intron using the position weight matrix (PWM) of our previous report on human consensus BPS [1]. For example, when a candidate BPS is "CTGAT", the sum of nucleotide probabilities at the five positions becomes 0.470 + 0.746 + 0.177 + 0.923 + 0.420 = 2.736. In a meantime, the best BPS is "CTCAT" with the sum of nucleotide probabilities of 3.007, whereas the worst BPS is "AGATG" with the sum of nucleotide probabilities of 0.360. PWM scores of the best and worst BPS are set to 1.000 and 0.000, respectively. Thus, the PWM score of "CTGAT" becomes 0.897.

Nucleotide probability at each position in human BPS [1]

Consensus	y	T	n	A	y
Position	-3	-2	-1	0	1
A	0.083	0.066	0.166	0.923	0.182
C	0.470	0.160	0.448	0.033	0.331
G	0.127	0.028	0.177	0.017	0.066
T	0.320	0.746	0.210	0.028	0.420

[c]Best-BPS-PPT, the best pair of BPS and PPT was determined according to the following algorithm. First, 'nYnAn' motif was looked for with an invariant 'A' at Int-50:Int-3 and set to be BPS_i. BPS_i located downstream of Int-9 was excluded because the length of PPT became less than 7 nucleotides. Second, the ratio of T/C's at positions +4 to +24 (PPT_i) from the invariant 'A' of BPS_i was calculated. This gave rise to multiple candidate BPS_i-PPT_i pairs at a single intron-exon boundary. The sum of the PWM of BPS_i and the T/C ratio in PPT_i was then calculated and a pair with the best sum score was selected.

[d]The exact motif for an RNA-binding protein was searched for at Int-50:Ex:Int+50 and scored according to SpliceAid 2 [2]. The sum of SpliceAid 2 scores was used as a feature for each RNA-binding protein.

References

1. Gao, K.; Masuda, A.; Matsuura, T.; Ohno, K. Human Branch Point Consensus Sequence Is YUnAy. *Nucleic Acids Res* **2008**, *36*, 2257–2267, doi:10.1093/nar/gkn073.
2. Piva, F.; Giulietti, M.; Burini, A.B.; Principato, G. SpliceAid 2: A Database of Human Splicing Factors Expression Data and RNA Target Motifs. *Hum Mutat* **2012**, *33*, 81–85, doi:10.1002/humu.21609.
