# Supplementary material for: FexSplice: A LightGBM-Based Model for Predicting the Splicing Effect of a Single Nucleotide Variant Affecting the First Nucleotide G of an Exon
Source: Genes (Basel). 2023 Sep 6;14(9):1765. doi: 10.3390/genes14091765 (PMC10531444; doi:10.3390/genes14091765)
Supplement: Supplementary file 1 [file genes-14-01765-s001.zip › Supplementary Table S3.pdf]

Supplementary Table S3. Comparison of 9 statistical measures of FexSplice, SpliceAI, and CI-SpliceAI

Model	FexSplice[b] (15-feature based LightGBM)	SpliceAI[c]	CI-SpliceAI[c]
**Accuracy[a]**	0.77 ± 0.07	0.54	0.58
**Precision[a]**	0.80 ± 0.12	0.85	0.82
**Recall[a]**	0.78 ± 0.13	0.22	0.22
**Specificity[a]**	0.77 ± 0.15	0.95	0.95
**F1 score[a]**	0.77 ± 0.07	0.35	0.34
**NPV[a]**	0.79 ± 0.11	0.49	0.55
**MCC[a]**	0.57 ± 0.15	0.24	0.25
**AUROC**	0.86 ± 0.08	0.69	0.65
**AUPRC**	0.87 ± 0.09	0.75	0.68

[a]Refer to Table 1 for the definitions of statistical measures.
[b]Statistical measures for FexSplice were calculated by 10-fold cross-validation (mean and SD) of our dataset, and are identical to those shown in Table 1.
[c]Statistical measures for SpliceAI and CI-SpliceAI are calculated by our dataset without cross-validation.
Note that FexSplice was trained with our dataset, whereas the others were not.
